# Percutaneous Endoscopic Gastrostomy Tube Placement in COVID-19 Patients

**DOI:** 10.3389/fnut.2021.603276

**Published:** 2021-06-04

**Authors:** Hemant Goyal, Aman Ali, Pardeep Bansal

**Affiliations:** ^1^The Wright Center for Graduate Medical Education, Scranton, PA, United States; ^2^Mercer University School of Medicine, Macon, GA, United States; ^3^The Commonwealth Medical College, Wilkes-Barre, PA, United States; ^4^Wilkes Barre General Hospital, Wilkes-Barre, PA, United States; ^5^Associate Digestive Care Associates, Kingston, PA, United States; ^6^Mercy Health - St. Vincent Medical Center, Toledo, OH, United States

**Keywords:** coronavirus, coronavirus (2019-nCoV), PEG tube, enteral feeding, endoscopy, SARS-CoV-2, COVID-19

## Abstract

Intensive care units (ICU) around the world are overburdened with COVID-19 patients with ventilator-dependent chronic respiratory failure (VDRF). Gastroenterology evaluations are being made to address the provision of chronic enteral feeding with the help of percutaneous endoscopic gastrostomy (PEG) placements in these patients. The placement of the PEG tube along with tracheostomy in patients with COVID-19 and prolonged VDRF may expedite discharge planning and increase the availability of ICU beds for other patients. Herein, we describe a multidisciplinary approach of PEG tube placements for patients with SARS-CoV-2-induced chronic VDRF for continued enteral feeding to avoid complications and decrease the length of stay.

## Key Points

- Careful patient selection with a good overall prognosis for PEG tube placement.
- A multidisciplinary team approach while considering patients' advance directives and family involvement.
- Local institutional guidelines should be followed regarding preprocedural COVID-19 testing.
- Use “cluster care” to reduce patient movements across different floors.
- Minimize the presence of personnel in the operating room to reduce exposure and save PPE.
- Proper donning and doffing of PPE.
- The “pull-technique” should be used while minimizing suctions to place the PEG tube.

Coronavirus Disease-2019 (COVID-19) caused by severe acute respiratory syndrome coronavirus-2 (SARS-CoV-2) has become a worldwide pandemic (1, 2). COVID-19 is primarily a pulmonary illness, although gastrointestinal manifestations are also common (3, 4). Intensive care units (ICU) worldwide are overburdened with COVID-19 patients who develop ventilator-dependent chronic respiratory failure (VDRF). These patients initially get enteral nutrition by nasogastric (NG) or orogastric (OG) tubes. However, there is a concern of local complications such as nasopharyngeal/oropharyngeal erosions and esophageal/gastric cardia ulcerations by the inserted feeding tube. An increased risk of chemical pneumonitis is also presumed due to the incomplete closure of the distal esophageal sphincter on prolonged NG/OG tube placements if the swallow mechanism remains impaired. Therefore, gastroenterology evaluations are called to address the provision of enteral feeding with the help of percutaneous endoscopic gastrostomy (PEG) tube placement (5). Although the presence of a PEG tube is also associated with its complications in the form of pneumonia, ileus, peristomal infection, and stomal leakage, nonetheless, continued enteral feeding gives a sense of relief to the patient's family and healthcare practitioners with a hope of improvement in the future (5). Moreover, placement of PEG-tubes along with tracheostomy in COVID-19 patients with VDRF may expedite discharge planning and can make the ICU beds available for other patients in the overburdened ICU during this pandemic. However, PEG-tube placement poses unique challenges for endoscopists and other endoscopy staff because of the potential of viral transmission, primarily due to aerosolization during suctioning (6, 7).

The nutritional management of critically ill COVID-19 patients is very similar to that of any other patient who develops VDRF. PEG-tube placement is a common procedure performed by endoscopists for continued enteral feeding in critically ill patients expected to recover. However, there are no gastrointestinal society recommendations for a maximum duration of NG/OG-tube in critically ill patients. Most physicians have a general consensus of about 2–3 weeks for the maximum duration of NG/OG-tube because of concerns of the increased risk of local complications after this duration, albeit unavailability of strong data about it. Currently, there are no available published data about the safety or feasibility of a PEG-tube in COVID-19 patients. Herein, we describe a multidisciplinary protocol for PEG-tube placements in COVID-19 patients regarding patient selection, the procedure timing, and safety precautions. This protocol may reduce anxiety among gastroenterologists with better placement rates, decrease complications, and hospital length of stay.

A careful patient selection of COVID-19 patients with VDRF is critical as the patients should have a reasonable chance of survival. If a PEG-tube placement is deemed necessary, then a multidisciplinary discussion should be held with the patient's family. The patients' known advance directives with onboard primary, procedural, and palliative care teams to discuss the goals of nutrition, overall prognosis, and risks and complications of the procedure itself should be considered. Many COVID-19 patients are on therapeutic anticoagulation because of the presence of venous thromboembolism (VTE) or increased risk of VTE. As per the American Society of Gastrointestinal Endoscopy (ASGE), PEG-tube placement is a high-risk procedure for bleeding complications, especially in patients on therapeutic anticoagulation (8). There is a paucity of data about the PEG tube placement in patients on therapeutic anticoagulation, including Coumadin and heparin. Therefore, patients' anticoagulation should be discontinued if they are being considered for PEG-tube placement. Another form of short-acting anticoagulation therapies (such as intravenous heparin or subcutaneous low molecular weight heparin) can be used while discontinuing them before the procedure as per the guidelines. Patients on aspirin only or clopidogrel alone are considered at low risk for bleeding from PEG tube placement, but there are no data regarding the dual antiplatelet therapy (8).

Guidelines from the COVID-19 tracheostomy task force recommend waiting at least 21 days before performing a tracheostomy (9). A similar window of 3 weeks for PEG-tube placement can be considered to avoid transmission if rapid SARS-CoV-2 testing is not available. Guidelines from the Joint Gastroenterological Society recommend rapid PCR-based testing within 48–72 h of the endoscopy wherever available (10). Most of these patients are critically ill due to COVID-19 and can have prolonged viral shedding on repeat nasal or nasopharyngeal testing (11). Therefore, though local institutional guidelines can be followed regarding pre-procedural COVID-19 testing, as a negative test might provide a sense of safety to the endoscopists and staff, a positive test should not affect the decision to place a peg tube or not. The decision should be made on clinical grounds for the requirement of peg tubes. In patients who tested positive, a timestamped method can be used to guide the endoscopy staff, and usually, critically sick patients will be considered non-infectious after 21 days and non-critically sick patients after 10 days. Standard precaution with proper donning and doffing should be used with these patients who tested positive. In facilities where COVID-19 testing is not available, a similar timestamped method can be used. A period of 21 days for critically sick patients and 10 days for non-critically sick patients could be used as an alternate safe point for PEG placement, but proper donning and doffing procedures should be followed as per institutional guidelines. The patients' movement along different floors should be avoided to reduce the transmission risk. However, if patients' transfer is deemed necessary, a plastic patient isolation drape can be used to prevent the spread while transporting patients from the ICU to the OR. The method of “cluster care” should be practiced, and PEG-tube placement should be planned immediately before or after tracheostomy to minimize patient transfer, limit contact, and save personal protective equipment (PPE).

Negative pressure OR should be used for the procedures. Only the minimum necessary staff (one GI nurse, one technician, and one nurse anesthetist), along with the endoscopist, should be present during the procedure to save PPE and reduce exposure. A small size (about 6 inches) can be made in the patient-isolation drape to access the expected site of PEG placement. All patients should receive prophylactic antibiotics as per the guidelines. The “pull-technique” should be used while minimizing suctions to place the PEG tube. In general practice, PEG-tube feeding can safely be started 4 h after tracheostomy/PEG tube placements, but this duration has varied for up to 24 h (11). Enteral feeding can be initiated with low-dose continuous enteral nutrition, advancing to full-dose continuous enteral nutrition within 48 h to meet the energy goal of 15–20 kcal/kg of actual body weight (12).

Overall, PEG-tube placement is a relatively safe procedure in appropriately selected COVID-19 patients who need chronic enteral tube feeding, with standard precautions outlined by national gastroenterological societies. With the development of institutional policies regarding patient selection and the timing of PEG placement with tracheostomy if needed, policies well-coordinated with the multidisciplinary team while minimizing endoscopic personnel during the procedure in COVID-19 patients can decrease the risk of transmission and improve patient care as well as free-up ICU resources.

## Author Contributions

HG: data collection and first draft. All authors conception and design, literature review, critical revision, and final approval of the manuscript.

## Conflict of Interest

The authors declare that the research was conducted in the absence of any commercial or financial relationships that could be construed as a potential conflict of interest.
